# The Impact of Clickers Instruction on Cognitive Loads and Listening and Speaking Skills in College English Class

**DOI:** 10.1371/journal.pone.0106626

**Published:** 2014-09-05

**Authors:** Zhonggen Yu, Wentao Chen, Yong Kong, Xiao Ling Sun, Jing Zheng

**Affiliations:** 1 School of Foreign languages of Hohai University, Nanjing City, Jiangsu Province, China, and School of English of Zhejiang Yuexiu University of Foreign Languages, Shaoxing City, Zhejiang Province, China; 2 School of English of Zhejiang Yuexiu University of Foreign Languages, Shaoxing City, Zhejiang Province, China; 3 School of Foreign languages of Hohai University, Nanjing City, Jiangsu Province, China; The University of Chicago, United States of America

## Abstract

Clickers might own a bright future in China if properly introduced although they have not been widely acknowledged as an effective tool to facilitate English learning and teaching in Chinese contexts. By randomly selecting participants from undergraduates in a university in China over four academic years, this study aims to identify the impact of clickers on college English listening and speaking skills, and differences in cognitive loads between clickers and traditional multimedia assisted instruction modes. It was concluded that in China's college English class, compared with multimedia assisted instruction, (1) clickers could improve college English listening skills; (2) clickers could improve college English speaking skills; and (3) clickers could reduce undergraduates' cognitive loads in College English Class. Reasons for the results and defects in this study were also explored and discussed, based on learning, teaching and cognitive load theories. Some Suggestions for future research were also raised.

## Introduction

English learning and teaching has been catching an increasing attention in China, especially in terms of listening and speaking. In order to communicate with foreigners, learners have to understand spoken English firstly and then respond in English. English learning and teaching of speaking and listening has been considered important for over two decades in China. Most of Chinese students are required to learn English in Grade Three in the primary school. They, however, still feel difficult to break through the threshold of English proficiency under traditional multimedia instruction. A number of college students complain that their listening and speaking skills remain unchanged though they have made great effort to practice for over one decade. They still feel hard to understand English broadcasting and speeches on TV. They also think it difficult for them to open their mouths to speak English full of confidence.

College students tend to be in a dilemma of heavy cognitive loads when attending English classes. They feel frequently bored with teachers' direct input of language points aided with traditional multimedia. Students also feel frightened when required to speak and listen to English through multimedia in class. Teachers often question students after listening and ask them to respond orally in English, which makes them nervous and anxious. Worse, teachers tend to allot a sea of assignment to students. Students, after painstakingly completing the assignment, still feel awkward and worried in class.

Clickers might own a bright future in China if properly introduced although they have not been widely acknowledged as an effective tool to facilitate English learning and teaching in Chinese contexts. Possibly influenced by the old tradition of keeping modest and cautious, Chinese college students tend to lower down their heads and keep silent when required to participate in peer discussion. After class, they frequently ask the teacher questions which are supposed to have been discussed among peers in class. The reasons why college students in China prefer to resort to the teacher after class may be of various kinds. Teachers are considered pundits in class, in whom students always believe. They may not believe in peers' answers because they think peers are on the same level of English as them. Peers may not know the answer either. Another reason may be that college students are worried about being silly if they ask peers some silly and laughable questions. Clickers, an anonymous polling device, may be able to overcome this worry although they are not so well-known and widely used in China as in the west.

Many studies were interested in use of Clickers in education of physics, science, engineering and mathematics [1]–[2]. This study, focused on clickers use in college English education, seems meaningful and innovative. This study aims to identify the impact of clickers on college English listening and speaking skills, and differences in cognitive loads between clickers and traditional multimedia assisted instruction modes. Corresponding to the research objectives, research questions are raised as: (1) can clickers improve college English listening skills compared with multimedia? (2) Can clickers improve college English speaking skills compared with multimedia? (3) Can clickers reduce undergraduates' cognitive loads in College English Class compared with multimedia? Three hypotheses are proposed as follows:

- Clickers can improve college English listening skills compared with multimedia;
- Clickers can improve college English speaking skills compared with multimedia;
- Clickers can reduce undergraduates' cognitive loads in College English Class compared with multimedia.

## Literature review

This section will review the past studies on use of clickers and cognitive loads. The role of clickers in cognitive loads will not be reviewed because so far, no studies on the role of clickers in cognitive loads have been carried out.

### Use of clickers

Many studies have explored the use of clickers in classes. The use of clickers in large-lecture introductory biology courses has been claimed to achieve success in learning outcomes [3]. Clickers have witnessed growing popularity in recent years, largely due to their role in encouraging all students to participate in lectures, particularly in large classes [4]–[8]. Several studies demonstrated that the use of clickers during lectures improved student performance on exams in undergraduate science classes [9]–[11].

Clickers were considered appealing to learners. It was shown by several studies that students enjoyed using clickers, felt that this form of interactive engagement was useful for their learning, and learned something from discussing questions with their peers in large-enrollment classes [12], [5], [9], [13]. With clickers, students will be encouraged to join peer discussion since they are supposed to vote anonymously. The anonymity may disperse their worry about silly mistakes they may make. The teacher is not considered the pundit in class and students are required to vote after discussion. This may be able to stimulate students' active participation into classroom activities even in China.

Instructors frequently couple peer instruction with clickers. The typical process of peer discussion integrated into clickers was: (1) the instructor raised a question and required students to answer; (2) students thought over the questions individually, after which they discussed with their peers; (3) students answered the question by voting through the electronic device; (4) if the majority of students voted wrongly, then they were required to re-vote after instructor's further explanation and peer discussion [14]. The instructor then showed histograms of student responses on the computer screen, which gave immediate feedback to both instructor and students on how well a concept was perceived.

### The cognitive load

Both the structure and characteristics of the cognitive architecture indicate that the primary purpose of instruction is to construct schemas in long-term memory and to minimize the limitation of working memory. Instructional designs that do not aim to alter long-term memory and that ignore working memory limitations when processing novel information are unlikely to be effective [15].

Besides human cognitive architecture, the cognitive load theory involves two distinct types of cognitive load [16]: intrinsic and extrinsic cognitive loads. The intrinsic cognitive load refers to the load embedded in the knowledge to be commanded, which depends on the ability to process the specific information simultaneously in working memory [17]–[18]. The intrinsic cognitive load is closely related to the degree of complexity of the target information and this load is hard to be altered. Cognitive load effects are not applied to the highly automatic information without learning objectives. An example is daily communication between family members which is not in need of complicated processing, and is easily perceived. This daily communication is significantly distinctive from the unfamiliar, complex, and advanced English language learning materials used in this study. The results obtained in this study will be only applicable to the materials which entail a heavy working memory load. The extrinsic cognitive load arises from instructional strategy which can be controlled and modified by the designer. It tends to be caused by an extrinsic increase in the elements which must be processed in working memory because of extra design in instruction. The majority of studies on the cognitive load focus on the decrease of extrinsic elements in instruction design that must be processed in working memory [19]–[20], [16].

Additionally, the term “germane cognitive load”, referred to as an independent cognitive load, is frequently used. Germane load is that load created in construction of schemas in learning and teaching [21]. It is claimed that because germane cognitive load depends on and assimilates the intrinsic cognitive load, it may be appropriate to define it within the category of intrinsic cognitive load [16]. Germane cognitive load refers to the resources occupying working memory in order to deal with the intrinsic cognitive load in learning process. Working memory is also required to process the extrinsic cognitive load. Reduction in the extraneous cognitive load can result in the increase of germane cognitive load, which can release working memory capacity for learning [16].

The measurement of mental cognitive load has caught the attention of educational psychologists recently. The attention has often been coupled with computer assisted learning [22]–[23]. The studies are often on the basis of the theoretical framework sourcing from cognitive load [23]–[24]. The cognitive load can be defined as a multidimensional construct representing the load that performs a particular task imposed on the learner's cognitive system. More specifically, the amount of cognitive load, measured at a given time, is a way of assessing the level of information being manipulated in working memory. Perception in the level of cognitive load or stress on working memory can help gauge the cognitive capacity for learning [21]. A rating scale, the NASA-Task Load Index (NASA-TLX), which consists of six component scales, is used in this study. An average of these six scales, weighted to reflect the contribution of each factor to the cognitive load of a specific activity from the perspective of the rater, is proposed as an integrated measure of overall cognitive load [25].

## Materials and Methods

The research lacks consent because the data were analyzed anonymously. The research has been approved by the authors' institutional review board–School of Foreign Languages of Hohai University, which waived the need for written informed consent from the participants.

This study adopted both qualitative and quantitative methods to identify the differences in college English listening and speaking skills and cognitive loads between multimedia and clickers instructions. This section will report the methods used in this study in terms of participants, instruments, and the procedure.

### Participants

The clickers experiment was conducted among undergraduates in a university in China who registered for college English ranging from the academic year 2009–2010 to 2012–2013. In the academic year 2009–2010, participants experienced traditional multimedia aided instruction in terms of college English. While during the academic years 2010 to 2011, 2011 to 2012, and 2012 to 2013, participants went through instruction aided with clickers. The registration was randomly operated without any gender bias or administrative regulation. The age of participants ranged from 17 to 22 years old.

### Instruments

The instruments used in this study include a CET4 listening test, a CET4 speaking test and a NASA-TLX 6-dimensional questionnaire, by which data were elicited (please see the Supporting Information “Dataset S1, Codebook S1 and Figure Data S1).

The first and second hypotheses were tested through examining the evolution of students' outcomes, where two types of data were involved: (1) means of listening scores in College English Test Band 4 (CET4); (2) the percentage rate of CET4 speaking scores over Grade C. Both data were calculated each academic year by the teaching administration of the University. To compare the results, we examined the data in the year when traditional multimedia instruction was used and the years when clickers were in use, i.e. the data sourced from academic years 2009–2010 (multimedia), 2010–2011, 2011–2012, and 2012–2013 (Clickers).

### The CET4 listening test

Listening tests in CET4 include three sections accounting for 249 points out of 710 in the whole CET4 including three sections.

### Section A

In this section, examinees heard 8 short conversations and 2 long conversations. At the end of each conversation, one or more questions were asked about what had been said. Both the conversation and the questions were spoken only once. After each question there was a pause. During the pause, examinees were required to read the four choices marked A), B), C) and D), and decide which was the best answer. Then they marked the corresponding letter on Answer Sheet 2 with a single line through the centre.

### Section B

In this section, examinees heard 3 short passages. At the end of each passage, they heard some questions. Both the passage and the questions were spoken only once. After examinees heard a question, they were required to choose the best answer from the four choices marked A), B), C) and D). Then they marked the corresponding letter on Answer Sheet 2 with a single line through the centre.

### Section C

In this section, examinees heard a passage three times. When the passage was read for the first time, examinees should listen carefully for its general idea. When the passage was read for the second time, examinees were required to fill in the blanks numbered from 36 to 43 with the exact words examinees had just heard. For blanks numbered from 44 to 46 examinees were required to fill in the missing information. For these blanks, examinees could either use the exact words examinees had just heard or write down the main points in their own words. Finally, when the passage was read for the third time, examinees should check what examinees had written.

### The CET4 speaking test

The results of CET4 speaking test are classified into following four grades.

- Grade A: Examinees are able to orally communicate in English regarding familiar issues with few difficulties.
- Grade B: Examinees are able to orally communicate in English regarding familiar issues with some difficulties.
- Grade C: Examinees are able to orally communicate regarding familiar issues using simple English.
- Grade D: Examinees are unable to communicate in English.

In the form of face-to-face communication, the speaking test is composed of three sections below.

- To answer examiners' questions such as self-introduction (5 minutes).
- To make personal speech (1.5 minutes) and join group discussion (4.5 minutes).
- To further answer examiners' questions (5 minutes).

### The NASA-TLX 6-dimensional questionnaire

The third hypothesis was tested by the NASA-TLX 6-dimensional questionnaire [25], which was designed to measure mental demand, physical demand, temporal demand, effort, frustration tolerance, and performance. Using NASA-TLX questionnaire, the cognitive load differences between multimedia and clickers instructions were identified by pre and post tests in the academic years 2009–2010 and 2010–2011. The questionnaire was rated by 5-point-Likert scale, ranging from “1 =  very much low/good” to “5 =  very much high/poor” (see Table 1).

**Table 1:** Description of NASA-TLX 6-dimensional questionnaire (Hart & Staveland, 1988).

Title	Endpoints	Descriptions	α
Mental demand	*Low /High*	How much mental and perceptual activity was required (e.g., thinking, deciding, calculating, remembering, looking, searching, etc.)? Was the task easy or demanding, simple or complex, exacting or forgiving?	.81
Physical demand	*Low /High*	How much physical activity was required (e.g., pushing, pulling, turning, controlling, activating, etc.)? Was the task easy or demanding, slow or brisk, slack or strenuous, restful or laborious?	.84
Temporal demand	*Low /High*	How much time pressure did you feel due to the rate or pace at which the tasks or task elements occurred? Was the pace slow and leisurely or rapid and frantic?	.80
Effort	*Low/High*	How hard did you have to work (mentally and physically) to accomplish your level of performance?	.88
Frustration level	*Low /High*	How insecure, discouraged, irritated, stressed and annoyed versus secure, gratified, content, relaxed and complacent did you feel during the task?	.88
Performance	*Good/poor*	How successful do you think you were in accomplishing the goals of the task set by the experimenter (or yourself)? How satisfied were you with your performance in accomplishing these goals?	.88

As shown in Table 1, the first column refers to categories of cognitive loads. The second one indicates the rating scale. The third one shows detailed questions for each cognitive load. The last one displays the values of Cronbach's alpha in the pilot study, which will be discussed in the following section.

### Procedure

A pilot study was firstly conducted to measure the internal reliability of the 5-point-Likert-scale questionnaire. Randomly selected participants (N = 43) joined the both pre and post tests and the results were entered into SPSS 13.0 to compute the Cronbach's alpha. The results showed that the questionnaire was internally reliable. Cronbach's alphas of mental demand, physical demand, temporal demand, effort, frustration level, and performance were .81, .84, .80, .88, .88, and .88 respectively (see Table 1).

The delivery of college English was mainly through traditional multimedia and information bulletin in the academic year 2009–2010 in the University. The lecturer taught students by presenting contents on a large screen connected to the multimedia projector and sometimes wrote language points and other related knowledge on the bulletin when needed. Students were asked to be ready to answer questions raised by the lecturer. They were required to preview what would be learnt before they attended the class, and review what they learned after class and finish the assignment allotted at home. Sometimes, there were quizzes in class for them to complete, which were considered as an important component of final scores.

The instruction model with clickers integrated face-to-face classroom learning into anonymous polling and peer discussion. The lecturer firstly selected a question from the bank and asked students to discuss with each other in English. After English-medium peer discussion, students were required to poll for the questions anonymously. The distribution of correct and incorrect responses would be displayed on the large screen in histograms immediately. If more than 70% students made correct responses, lecturers would continue to the next topic after simple explanation. If less than 30% students provided the correct answers, then lecturers would stop to further explain the topic in detail and afterwards asked students to discuss and poll once more until more than 70% students made correct choices. If the correct percentage rate fell between 70% and 30%, students were required to discuss for the second polling. It should be empathized that all the responses made by students were anonymous. Even though they made any ridiculous or stupid choices, others would not know. This relaxed students and made them more actively participate in classroom activities.

At the very beginning of the semester, lecturers introduced the new model of teaching and emphasized the important role of clickers and peer discussion as essential complements to classroom learning. Students were encouraged to participate in peer discussion in English and would be given a bonus if they kept a high frequency of polling and peer discussion. Otherwise, they would be punished via negative valuation on their performance. In case students were absent, lecturers could easily spot them out through polling since clickers could easily identify students' demographic information through polling. This could also increase students' attendance.

To successfully complete this mode with clickers, the University financially supported it through a teaching innovation project. One experienced professor was in charge of the project, and four lecturers who had been teaching college English for over five years participated in the innovation project. Every week, lecturers checked whether the project was correctly and smoothly carried out. Every month, the professor gathered lecturers to check the progress and address problems. Students with different performances were also irregularly invited to talk in order to keep everything on track. In order to minimize the possible bias, the final exams were randomly distributed among lecturers to review.

Of the total of 1142 students who registered for college English, during the academic year 2009–2010, the questionnaire was randomly distributed to the 1021 students who sat the final exam. Totally, 1016 filled questionnaires were gathered, among which 174 were invalid due to incomplete information, unanimous answers and unclear replies. Consequently, 842 valid questionnaires were taken into serious consideration and considered as a sample representing the population. During the academic year 2010–2011 when clickers were in process and embedded in students and lecturers' mind, the same questionnaires were also distributed to 1021 students who registered for the course college English. Finally, the valid questionnaires were 763 except the invalid.

## Results

Results of this study will be reported strictly based on the sequence of hypotheses raised.

### Hypothesis 1: Clickers can improve college English listening skills compared with multimedia

**Figure 1:**
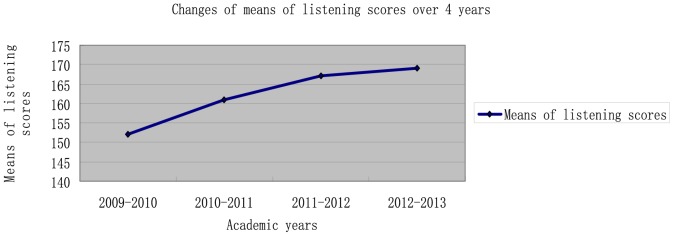
Changes of means of listening scores over 4 years.

To test this hypothesis, means of listening scores over 4 academic years were calculated by the teaching administration of the University, and entered into EXCEL for graphing (Please see figure 1).

As shown in Figure 1, means of listening scores in the academic year 2009–2010 (multimedia) are lower than those in other academic years (clickers). Starting from the mean around 150, the means sharply go up from 2009–2010 to 2010–2011, and keep significantly increasing from 2010–2011 to 2011–2012. The academic year 2012–2013 witnesses a steady rise in the mean. This result indicates that clickers are able to improve participants' listening skills compared with multimedia. Therefore, the hypothesis “Clickers can improve college English listening skills compared with multimedia” is accepted.

### Hypothesis 2: Clickers can improve college English speaking skills compared with multimedia

**Figure 2:**
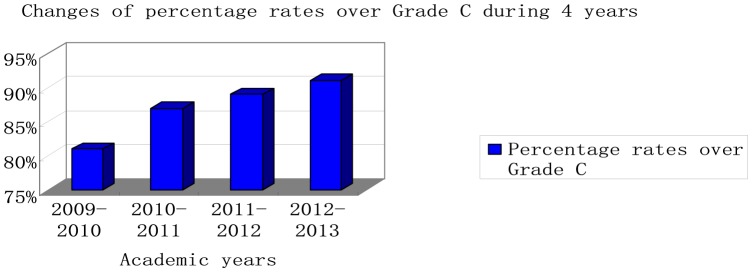
Changes of percentage rates over Grade C during 4 years.

To test this hypothesis, the percentage rate of undergraduates who obtained the results over Grade C was calculated. (The percentage rate  =  the number of participants who scored over Grade C/the number of participants who joined the CET4 speaking test). Figure 2 shows the changes of this rate over 4 academic years.

As revealed in Figure 2, the percentage rate over Grade C of CET4 speaking test in 2009–2010 (multimedia) is lower compared with other academic years when clickers are in use. From 2009–2010 to 2010–2011, there is a sharp rise in the percentage rate, while the other academic years witness a continuously steady increase in percentage rates of CET4 speaking tests. This implies that clickers realize students' increase in speaking skills compared with multimedia. Consequently, the hypothesis “Clickers can improve college English speaking skills compared with multimedia” is accepted.

### Hypothesis 3: Clickers can reduce undergraduates' cognitive loads in College English Class compared with multimedia

The cognitive load was measured in terms of the NASA-TLX 6-dimensional questionnaire [25] between randomly selected 49 participants in the academic year 2009–2010 (traditional multimedia) and 56 participants in the academic year 2010–2011 (clickers). After removing the incomplete and invalid information, 42 and 47 questionnaires in multimedia and clickers instruction were selected respectively. The data was processed in the program One-Way ANOVA in SPSS 13.0 summarized in Table 2.Table 2 reveals significantly lower cognitive loads under clickers instruction than multimedia. As described in Table 2, participants instructed by way of traditional multimedia show significantly higher mental demand than those instructed by clickers (F = 8.52, p = .004). This indicates that the instruction with clickers significantly require less mental and perceptual activity than multimedia instruction. With clickers, the task is considered easier or less demanding than with multimedia. Physical demand is significantly less in clickers instruction than in multimedia (F = 9.43, p = .003), which demonstrates that less physical activity was required in clickers instruction than multimedia and that the task is easier or less demanding with clickers than multimedia. Under clickers instruction, temporal demand is significantly less than multimedia (F = 9.84, p = .002). This shows that with clickers participants feel less time pressure due to the rate or pace at which the tasks or task elements occur than with multimedia and they also feel that the pace is slower and more leisurely with clickers than with multimedia. The effort made by participants with clickers is significantly less than those with multimedia (F = 11.83, p = .001). This refers to the likelihood that participants under clickers instruction work less laboriously to accomplish the level of performance than those under multimedia instruction. The frustration level under clickers instruction is significantly lower than under multimedia instruction (F = 12.89, p = .001). This means that participants under clickers instruction feel significantly less insecure, discouraged, irritated, stressed and annoyed than those under multimedia instruction. Participants under clickers instruction outperform those under multimedia instruction (F = 12.22, p = .001). This implies that participants under clickers instruction think they are more successful in accomplishing the goals and more satisfied with their performance in accomplishing these goals compared with those under multimedia instruction.

**Table 2:** Differences in cognitive loads between multimedia and clickers instruction.

Dimensions	Instruction models	N	Mean	Std. Deviation	F	Sig.
Mental demand	Multimedia	42	3.88	.54	8.52	.004
	Clickers	47	3.53	.58		
Physical demand	Multimedia	42	3.87	.53	9.43	.003
	Clickers	47	3.51	.57		
Temporal demand	Multimedia	42	3.87	.54	9.84	.002
	Clickers	47	3.48	.59		
Effort	Multimedia	42	3.87	.52	11.83	.001
	Clickers	47	3.47	.57		
Frustration level	Multimedia	42	3.85	.52	12.89	.001
	Clickers	47	3.44	.55		
Performance	Multimedia	42	3.12	.35	12.22	.001
	Clickers	47	3.49	.60		

The results show that the cognitive load under clickers instruction is significantly less than under multimedia. Thus, the hypothesis “Clickers can reduce undergraduates' cognitive loads in College English Class compared with multimedia” is accepted.

## Discussion

This section will explore the reasons for the findings in this study, coupled with some features of clickers.

### Peer discussion

A prominent advantage of clickers is the ability to encourage peer discussion. Clickers and the designed questions tend to be used coupled with peer discussion, which is a key assistant tool to maximize the effectiveness of use of clickers. This tool encourages students to voice their ideas and discuss with their peers to reach an agreement [14]. Studies investigating the undergraduate students showed that students were more likely to answer a question correctly after peer discussion compared with those without peer discussion [13]–[14], [26]. Furthermore, studies that used pairs of matched questions revealed that students learned from discussing clickers questions with their peers [13], [27] and this peer-based interaction was especially effective when it was followed by instructor's further explanation [26].

Peer discussion might also have caught participants' attention. Peers might tend to concentrate on the topic in peer discussion. They, however, might be easily distracted by teachers' tedious delivery. Speakers might have been encouraged to voice their opinions by peers and peers might have been interested in the more confident speeches urged by themselves. This could doubtlessly make peer speech more easily understood and listeners more willing to hear. Thus a speaking-listening benign cycle might have formed, which led to improvements on both listening and speaking.

### The reduction of language anxiety

Another noteworthy advantage of clickers might be the role in the reduction of language anxiety. Language anxiety could negatively influence foreign language learning through interfering with the “acquisition, retention, and production of the new language” [28]. Many students feel more anxious in a foreign language class than other classes [29]. Anxious students usually sit at the back of the classroom and feel unwilling to participate in classroom activities. They always attempt to avoid the duty of doing assignment and tend to be involuntary to orally answer questions [28]. They also try to avoid complicated sentences when orally answering questions due to less confidence [30].

Clickers can realize anonymous polling and encourage students to join peer discussion. Students may feel relaxed when they discuss with peers rather than teachers, and they may also be less anxious when they answer questions through anonymous polling. The reduction in anxiety might have pushed students who preferred to sit at the back to move their seats forward and turned their unwillingness into willingness. In this way, with less anxiety, students might have practiced speaking English and listening to their peers with more interest. It is thus unsurprising that with clickers participants' listening and speaking skills improved more greatly than with multimedia.

### Anonymous polling

Polling with anonymity cannot be neglected even a little when the effectiveness of clickers is explored. With clickers, participants could poll anonymously and they do not need to worry about any silly mistake. They might have become more active in class than multimedia instruction which needs face-to-face response to questions. Participants' active participation into classroom activities, such as peer discussion and polling, might have increased the opportunities for them to speak and listen to English in class. The more they speak and listen, the more progress they will make. The long-term instruction with clickers might make participants cultivate a habit of communicating with peers in English. This habit is definitely beneficial for their English learning.

Anonymous polling might also subconsciously stimulate participants to think in English. Thinking in English might help them to organize ideas in a more rapid and systematic way, which would facilitate their oral expression. In peer discussion, participants might suggest some inappropriate pronunciations and erroneous expressions for their peers. Peers might be readier to accept peers' suggestions than lecturers'. This interaction could possibly have consolidated their speaking skills.

### Motivation

From the academic year 2009–2010 (multimedia) to 2010–2011, participants' speaking and listening skills sharply rose. This might indicate that participants felt excited and thus motivated when firstly instructed with clickers. The motivation might have improved their listening and speaking skills to a large extent. Afterwards, the listening and speaking skills only improve steadily. This might imply that participants were accustomed to the clickers assisted teaching style and remained less excited and motivated. However, their listening and speaking still remained more proficient than those under traditional multimedia teaching. This might prove that clickers could improve listening and speaking skills at the present level of technology and teaching style. However, if clickers were continuously developing and ceaselessly bringing surprise or excitement to students, students' speaking and listening skills might rocket further.

### Receptive and productive

Listening belongs to a receptive skill, which is passive in learning process. Participants were passively waiting for linguistic input in class in order to improve listening skills. Clickers teaching might have provided this opportunity by activating participants to discuss topics, which prepared listening materials for the passive listeners. By contrast, speaking is classified into a productive skill, which is an active demand for learners, needing students to open their mouths to utter sentences. Clickers teaching might have encouraged this activity via peer discussion. Students might have been more relaxed when faced with peers than when confronted with the lecturer and the whole class in multimedia teaching.

### The cognitive load

In peer discussion, the complexity of the topic might be dispersed and weakened among groups. The intrinsic cognitive load which is closely related to the degree of complexity of the target information might be decreased. Under multimedia instruction, lecturers tend to design the delivery pattern and require students to follow. This might lead to an extrinsic increase in the elements which must be processed in working memory because of extra design in instruction. However, under the instruction with clickers, it is mainly students who influence the pattern of teaching and learning. They discuss and poll and lecturers select the teaching progress based on the results. This might decrease extrinsic elements in instruction design that must be processed in working memory and thus the extrinsic load might be released. Reduction in the extraneous cognitive load might increase germane cognitive load, which might release working memory capacity for learning [16]. Learning efficiency might thus be heightened and the learning and teaching process might be optimized.

Admittedly, although clickers have been proved effective to improve listening and speaking skills and to release cognitive loads, they might still have disadvantages. In a small-size class, the lecturer might feel more convenient and more time saving through traditional multimedia presentation than through clickers. Financial expense might also be an unavoidable factor to be considered.

## Conclusion

This part will discuss both advantages and disadvantages of this study, together with suggestions for future research.

### Advantages

This study, combining qualitative with quantitative research methods and arriving at conclusions consistent with previous studies, seems convincing and worth reading. The sample size, over several thousand, is considered large enough to represent the population. The study looked at the changes of cognitive loads, listening and speaking skills among thousands of participants over four academic years. As an instrument to identify learners' English proficiency, CET4 is organized by the Ministry of Education of China and has been proved valid and reliable to test students' English proficiency in terms of listening, speaking, reading and writing since it was established in 1987 [31]. The NASA-TLX 6-dimensional questionnaire, since designed by Hart and Staveland in 1988, has also experienced numerous experiments conducted by scholars and been proved internally reliable and valid to measure cognitive loads. The outstanding merit of this study is that it explores the effectiveness of clickers in college English rather than in science and engineering. The majority of previous studies focused on science and engineering, ignoring the field of arts and humanities, let alone listening and speaking skills of English as a foreign language. This study compensates for this regret.

### Disadvantages

Nevertheless, there are also deficits in this study. Different lecturers have different teaching styles and various teaching capacities even when they use the same clickers system. During the four academic years, students might have been situated in different learning and teaching environments and received different styles of instruction. The scorers for each test paper might hold different attitudes towards each student's answers. All of these might be “extrinsic or intrinsic” factors influencing the results in the study.

### Prospects

Future studies on use of clickers in class might center more on arts and humanities than on science and engineering since much discussion and exploration has been developed in science and engineering. More studies might be needed in the east rather than in the west since many studies have been carried out in the west. Integration between teaching, learning, cognition, neurology, and psychology might also be necessary since learning and teaching cannot develop alone without cross-disciplinary cooperation. It is also necessary for clickers manufacturers to update the equipment in order to produce more advanced and less expensive products. The integration of academic research on use of clickers into advancements of educational technologies might help improve teaching and learning effectiveness.

## Supporting Information

Figure Data S1 **Changes of percentage rates over Grade C during 4 years.**
(XLS)

Dataset S1 (RAR)

Codebook S1 (RAR)
